# Mental health symptoms and associated factors among primary healthcare workers in China during the post-pandemic era

**DOI:** 10.3389/fpubh.2024.1374667

**Published:** 2024-05-14

**Authors:** Difei Liu, Yuhe Zhou, Xubowen Tao, Yutong Cheng, Rui Tao

**Affiliations:** ^1^School of Education, Hefei University, Hefei, China; ^2^School of Physical Education, Anhui Normal University, Wuhu, China; ^3^Department of Psychiatry, Chaohu Hospital of Anhui Medical University, Hefei, China; ^4^Department of Psychiatry, School of Mental Health and Psychological Sciences, Anhui Medical University, Hefei, China; ^5^Department of Psychiatry, Anhui Psychiatric Center, Hefei, China

**Keywords:** primary healthcare workers, mental health, associated factors, the post-pandemic era, China

## Abstract

**Background:**

The impact of the COVID-19 pandemic on mental health among healthcare workers has been widely reported during the initial and ongoing phases of the COVID-19 pandemic. Yet, little remains known about the mental health status of primary healthcare workers in China during the post-pandemic era.

**Methods:**

A cross-sectional study was conducted between March 1, 2023, and May 31, 2023 in Anhui China. A total of 13,530 primary healthcare workers were recruited. Multiple logistic regression was used to identify potential factors associated with the incidence of depression and anxiety among primary healthcare workers.

**Results:**

The prevalence of depression and anxiety among primary healthcare workers was 50.7 and 26.4%, respectively. Multiple logistic regression revealed that female gender (OR = 1.345, 95%CI = 1.222–1.479), being divorced or widowed (OR = 1.432, 95%CI = 1.128–1.817), being a nurse (OR = 1.250, 95%CI = 1.126–1.388), and working more than 8 h per day (OR = 1.710, 95%CI = 1.583–1.846) were significantly associated with depression. A higher risk of anxiety among primary healthcare workers was associated with female gender (OR = 1.338, 95%CI = 1.198–1.494), being divorced or widowed (OR = 1.373, 95%CI = 1.056–1.770), being a nurse (OR = 1.139, 95%CI = 1.013–1.282), and working more than 8 h per day (OR = 1.638, 95%CI = 1.497–1.794). Better monthly income, more than 21 years of working experience and without experience of workplace violence were protective factors against depression and anxiety during the post-pandemic era.

**Conclusion:**

Depressive symptoms are more common among primary healthcare workers in China during the post-pandemic era. Female gender, being divorced or widowed, being a nurse, working years, working seniority, monthly income, and experience of workplace violence were identified as associated factors. Targeted intervention is needed when developing strategies to reduce depression and improve primary healthcare workers’ wellness and mental health.

## Introduction

The COVID-19 pandemic has created a significant global challenge, and the impact of the pandemic on public health has been widely reported (1–6). Many individuals are experiencing increased levels of depression, anxiety, stress, insomnia, post-traumatic stress, fear, and burnout during the initial and ongoing phases of the COVID-19 pandemic (7–13). Previous studies on this topic focused on the acute effects of the COVID-19 pandemic on psychological symptoms among the general population (anxiety: 25.6%, depression: 23.1%) (14) and healthcare workers (anxiety: 36.2%, depression: 50.4%) (15) in different countries. A large-scale study revealed that during the first wave of the COVID-19 pandemic, 33.0 and 46.6% of the general public living in Wuhan China (the city experiencing the most severe COVID-19 outbreak), experienced anxiety and depressive symptoms (16). A systematic review and meta-analysis to estimate the prevalence of depression and anxiety related to COVID-19 among affected general populations were 15.97 and 15.15%, respectively (17), while a meta-analysis included 401 studies, representing 458,754 healthcare workers across 58 countries suggested that the prevalence of depression was 28.5%, anxiety was 28.7%, and insomnia was 24.4% (18). It can be seen that the epidemic has had a huge impact on the whole population, especially healthcare workers (13, 19).

Mental health symptoms of healthcare workers have become a significant public problem in the healthcare system during the COVID-19 pandemic (20–22). Healthcare workers have concerns about the risk of COVID-19, inadequate personal protective equipment, excessive workload, workplace violence, and many more easily experience mental health disturbances (23). Depression, particularly anxiety, increased in healthcare workers from the beginning to the COVID-19 pandemic peak (24). Martín-del-Campo F et al. found that the severity of anxiety increased immediately after the onset of the COVID-19 pandemic, and then decreased over time (25), while posttraumatic stress symptoms of healthcare workers increased over time during the COVID-19 pandemic (20). Depressive symptoms may persist well after the pandemic, and such problems are often precursors of psychiatric disorders. Healthcare workers who develop depressive disorder during COVID-19 may be at greater risk for long-term adverse outcomes. Therefore, it is important to investigate the impact of the long-term effects of the COVID-19 pandemic on the mental health status of healthcare workers.

In China, primary healthcare workers play a vital role as the “first line of defense” in protecting public life and health. They have to deal with pre-screening, referrals, public awareness of the epidemic, and also ensure that basic health services are available for other diseases. The physical and psychological burden on primary healthcare workers has significantly increased (26, 27). In addition to the unreasonable allocation of medical resources, primary healthcare workers have faced increased workplace violence during the COVID-19 pandemic (28, 29). To the best of our knowledge, most existing studies have focused on frontline healthcare workers in COVID-19 designated hospitals (30, 31), while overlooking primary health workers involved in epidemic prevention and control in primary hospitals. We have very limited information regarding the changes in the psychological well-being of these primary health workers after China lifted its social blockade and epidemic control measures on January 8, 2023. Moreover, psychiatric symptoms among healthcare workers can persist long after a pandemic, often serving as a precursor to mental illness and potentially leading to long-term adverse outcomes. Paying attention to the mental health status of primary health workers in the post-pandemic era is important for implementing targeted intervention measures. Therefore, it is of great significance to investigate the mental health status in the post-pandemic era.

## Methods

### Study design, setting, and participants

To investigate depression and anxiety among primary healthcare workers and identify associated factors during the post-pandemic era, an online survey was conducted among primary healthcare workers working in Anhui China. This cross-sectional study utilized the random whole cluster sampling method and was active between March 1, 2023, and May 31, 2023. This timeframe coincided with the lifting of societal lockdown in China. There are 138 county hospitals in 59 counties of Anhui Province. Firstly, 25 counties were randomly selected from the 59 counties of Anhui Province, and then one county hospital was randomly selected from each of the 25 counties to participate in the survey. The study targeted the 25 county hospitals in Anhui Province, and primary healthcare workers from these hospitals were invited to participate in the survey. Finally, the study targeted the 25 county hospitals in Anhui Province, and primary healthcare workers from these hospitals were invited to participate in the survey. All participants read and agreed to the informed consent form before the survey, which stated the importance, objectives, voluntariness, and confidentiality principles of the survey. Before volunteers filled out the online questionnaires via mobile phone or computer, they were informed that they had the right to withdraw at any time. To achieve the most honest answers, three pairs of the same questions were set in the questionnaire. Questionnaires with inconsistent responses were excluded, as well as questionnaires with a response time of less than 45 s and questionnaires with missing questions. Finally, a total of 12,764 primary healthcare workers (*n* = 13,530) were included in the statistical analyses, and the valid response rate was 94.3%.

In this cross-sectional study, socio-demographic characteristics (gender, age, educational level, marital status), work-related variables (technical post title, profession, monthly income, work seniority, daily work hours, experience of workplace violence), and mental health variables (depression and anxiety) were collected using this online questionnaire. The Patient Health Questionnaire (PHQ-9) was used to investigate the depressive symptoms of primary healthcare workers, and the 7-item Generalized Anxiety Disorder Scale (GAD-7) was used to investigate their anxiety. The research protocol was approved by the Ethics Committee of Chaohu Hospital of Anhui Medical University.

### Questionnaire

Anxiety symptoms were measured using clinically validated scales for GAD-7, which evaluate the frequency of anxiety symptoms over the past 2 weeks (32, 33). The total score ranges from 0 to 21, with a high score indicating a greater severity of anxiety. The presence of anxiety was defined as a GAD-7 score ≥ 5, with scores of 5–9 indicating mild anxiety symptoms, scores of 10–14 indicating moderate anxiety symptoms, and scores of ≥15 indicating severe anxiety symptoms. The Chinese GAD-7 scale has been widely used in previous studies. The Cronbach’s α coefficient for this study was 0.942.

PHQ-9, which provides a reliable and valid measure for depressive symptoms over the past 2 weeks, has been used in different settings (27, 34, 35). It consists of 9 items, and each item is answered on a 4-point Likert-type scale, with scores ranging from 0 (“never”) to 3 (“nearly every day”). The PHQ-9 total score ranges from 0 to 27, and a higher total score indicates greater severity of depression. A total score of ≥5 represents the presence of depressive symptoms, with scores of 5–9 indicating mild depression, scores of 10–14 indicating moderate depression, scores of 15–20 indicating moderate to severe depression, and scores of ≥20 indicating severe depression. The Cronbach’s alpha of the PHQ-9 in our study was 0.909, which demonstrates good reliability.

### Statistical analysis

Statistical analysis was performed with IBM SPSS Statistics version 25.0. The sample distribution was conducted using frequency for categorical variables and mean ± standard deviation for continuous variables. For the statistical analysis, the Chi-square test was utilized to assess variables that were not in a normal distribution. The independent correlates of depression and anxiety were examined through multiple logistic regressions, with the symptoms of depression or anxiety as the dependent variables (yes, no). Gender (male and female), educational level (associate degree, bachelor degree, and master degree or more), marital status (single, married, and divorced/widowed), technical post title (junior title, intermediate grade, and senior title), profession (doctor, nurse, and medical technicians), income (≤ 3,000 RMB, 3001–8,000 RMB, ≥8,001 RMB), work seniority (≤10 years, 11–20 years, ≥21 years), daily work hours (≤8 h, >8 h), and experience of workplace violence(yes, no) were entered as the independent variables. The level of statistical significance was set at *p*-values of 0.05 (two-tailed).

## Results

### Socio-demographic characteristics of primary healthcare workers in China

The socio-demographic characteristics of Chinese primary healthcare workers were shown in Table 1. The mean age of participants was 37.2 years old. 8,829 participants (69.2%) were female, 6,029 (47.2%) were under the age of 34, and 10,217 (80.0%) were married. 7,530 (59.0%) participants had a bachelor degree or more, 6,952 (54.5%) had a junior title. 4,072 participants (31.9%) were doctors, 5,717 (44.8%) were nurses, and 2,975 (23.3%) were medical technicians. Additionally, 8,245 participants (64.6%) reported working more than 8 h per day. Of all the participants, 8,442 (66.1%) had no experience of workplace violence.

**Table 1:** Socio-demographic characteristics of primary healthcare workers (*n* = 12,764).

Variable	Participants	Depression		Anxiety		Depression	Anxiety
	*n* = 12,764	Yes (*n* = 6,466)	No (*n* = 6,298)	Yes (*n* = 3,367)	No (*n* = 9,397)	χ^2^ (*p*-value)	χ^2^ (*p*-value)
*Gender (N, %)*							
Male	3,935 (30.8)	1796 (27.8)	2,139 (34.0)	899 (26.7)	3,036 (32.3)		
Female	8,829 (69.2)	4,670 (72.2)	4,159 (66.0)	2,468 (73.3)	6,361 (67.7)	57.272 (<0.001)	36.556 (<0.001)
*Age (N, %)*							
*≤34*	6,029 (47.2)	3,122 (48.3)	2,907 (46.2)	1712 (50.8)	4,317 (45.9)		
*35–44*	3,750 (29.4)	1939 (30.0)	1811 (28.8)	1,007 (29.9)	2,743 (29.2)		
≥45	2,985 (23.4)	1,405 (21.7)	1,580 (25.1)	648 (19.2)	2,337 (24.9)	20.088 (<0.001)	46.593 (<0.001)
*Educational level (N, %)*							
Associate degree	5,234 (41.0)	2,528 (39.1)	2,706 (43.0)	1,242 (36.9)	3,992 (42.5)		
Bachelor degree	7,176 (56.2)	3,756 (58.1)	3,420 (54.3)	2025 (60.1)	5,151 (54.8)		
Master degree or more	354 (2.8)	182 (2.8)	172 (2.7)	100 (3.0)	254 (2.7)	19.861 (<0.001)	32.068 (<0.001)
*Marital status (N, %)*							
Married	10,217 (80.0)	5,157 (79.8)	5,060 (80.3)	2,638 (78.3)	7,579 (80.7)		
Single	2,246 (17.6)	1,132 (17.5)	1,114 (17.7)	635 (18.9)	1,611 (17.1)		
Divorced or widowed	301 (2.4)	177 (2.7)	124 (2.0)	94 (2.8)	207 (2.2)	8.188 (0.017)	9.438 (0.009)
*Technical post title (N, %)*							
Junior title	6,952 (54.5)	3,448 (53.3)	3,504 (55.6)	1792 (53.2)	5,160 (54.9)		
Intermediate grade	4,676 (36.6)	2,452 (37.9)	2,224 (35.3)	1,274 (37.8)	3,402 (36.2)		
Senior title	1,136 (8.9)	566 (8.8)	570 (9.1)	301 (8.9)	835 (8.9)	9.373 (0.009)	3.114 (0.211)
*Profession (N, %)*							
Doctor	4,072 (31.9)	1959 (30.3)	2,113 (33.6)	1,029 (30.6)	3,043 (32.4)		
Nurse	5,717 (44.8)	3,117 (48.2)	2,600 (41.3)	1,664 (49.4)	4,053 (43.1)		
Medical technicians	2,975 (23.3)	1,390 (21.5)	1,585 (25.2)	674 (20.0)	2,301 (24.5)	63.159 (<0.001)	45.712 (<0.001)
*Income (N, %)*							
≤3,000	1848 (14.5)	966 (14.9)	882 (14.0)	521 (15.5)	1,327 (14.1)		
3,001–8,000	10,265 (80.4)	5,203 (80.5)	5,062 (80.4)	2,683 (79.7)	7,582 (80.7)		
≥8,001	651 (5.1)	297 (4.6)	354 (5.6)	163 (4.8)	488 (5.2)	8.536 (0.014)	4.041 (0.133)
*Work seniority (N, %)*							
≤10	5,502 (43.1)	2,814 (43.5)	2,688 (42.7)	1,543 (45.8)	3,959 (42.1)		
11–20	4,239 (33.2)	2,250 (34.8)	1989 (31.6)	1,183 (35.1)	3,056 (32.5)		
≥21	3,023 (23.7)	1,402 (21.7)	1,621 (25.7)	641 (19.0)	2,382 (25.3)	32.615 (<0.001)	54.642 (<0.001)
*Work hours per day (N, %)*							
≤8	4,519 (35.4)	1893 (29.3)	2,626 (41.7)	903 (26.8)	3,616 (38.5)		
>8	8,245 (64.6)	4,573 (70.7)	3,672 (58.3)	2,464 (73.2)	5,781 (61.5)	251.181 (<0.001)	147.393 (<0.001)
*Experience of workplace violence (N, %)*							
No	8,442 (66.1)	3,888 (60.1)	4,554 (72.3)	1879 (55.8)	6,563 (69.8)		
Yes	4,322 (33.9)	2,578 (39.9)	1744 (27.7)	1,488 (44.2)	2,834 (30.2)	211.301 (<0.001)	218.031 (<0.001)

6,466 primary healthcare workers (50.7%) were classified as having depressive symptoms (a score of PHQ-9 ≥ 5), and 3,367 primary healthcare workers (26.4%) were classified as having anxiety symptoms (a score of GAD-7 ≥ 5). Significant differences were found between primary healthcare workers with and without depression and anxiety symptoms in relation to the demographic variables as shown in Table 1.

### Prevalence of depression and anxiety among primary healthcare workers

More than two-thirds (72.2%) of primary healthcare workers with depression were female, and 73.3% of primary healthcare workers with anxiety were female. The prevalence of depression in primary healthcare workers was 50.7%, and there was a significant gender difference (45.6% in males and 52.9% in females, *p* < 0.001). The prevalence of anxiety in primary healthcare workers was 26.4%, and there was a significant gender difference (22.8% in males and 28.0% in females, *p* < 0.001). Compared to those with bachelor degree or more (depression: bachelor degree 52.3%, master degree or more 51.4%; anxiety: bachelor degree 28.2%, master degree or more 28.2%), those with an associate degree (depression: 48.3%; anxiety: 23.7%) had significantly lower rates of depression and anxiety (both *p* < 0.001). The incidence of depression in primary healthcare workers who were divorced/widowed was higher than those who were married or single (*p* < 0.01). The incidence of anxiety in primary healthcare workers who were married was lower than those who were divorced/widowed or single (both *p* < 0.05). Compared to primary healthcare workers who were more than 45 years old (depression: 47.1%; anxiety: 21.7%), those who were less than 45 years old had a significantly higher incidence of depression and anxiety (both *p* < 0.001).

Compared to doctors and medical technicians (depression: doctor 48.1%, medical technicians 46.7%; anxiety: doctor 25.3%, medical technicians 22.7%), there was a significantly higher incidence of depression and anxiety in nurses (depression: 54.5%, anxiety: 29.1%, both *p* < 0.001). The prevalence of depression and anxiety in primary healthcare workers who work over 8 h per day was higher than those who work less than 8 h per day (depression: 55.5% vs. 41.9%; anxiety: 29.9% vs. 20.0%, all *p* < 0.001). The prevalence of depression among primary healthcare workers with low and medium (≤8,000 RMB) income was higher than those with high income (≥8,001 RMB), while there was no difference between low income and medium income. There was no significant difference in the prevalence of anxiety among different income groups. Compared to primary healthcare workers with no experience of workplace violence (depression: 46.1%, anxiety: 22.3%), those with experience of workplace violence (depression: 59.6%, anxiety: 34.4%) had a significantly higher prevalence of depression and anxiety (both *p* < 0.001) (Table 1).

### Factors associated with depression and anxiety in multiple logistic regressions

We performed multiple logistic regression analyses to examine the associations between depression and other factors. The references of the categorical variables were defined as shown in Table 2. The results showed that female gender (OR = 1.345, 95%CI = 1.222–1.479), being divorced or widowed (OR = 1.432, 95%CI = 1.128–1.817), being a nurse (OR = 1.250, 95%CI = 1.126–1.388), and working more than 8 h per day (OR = 1.710, 95%CI = 1.583–1.846) were associated factors for depression among primary healthcare workers. Medium (OR = 0.860, 95%CI = 0.774–0.956) and high monthly income (OR = 0.672, 95%CI = 0.549–0.823), and without experience of workplace violence (OR = 0.558, 95%CI = 0.515–0.604) were protective factors.

**Table 2:** Multiple logistic regression examining individual characteristics associated with depression in primary healthcare workers.

Variables	B	*p*-value	OR	95% CI
*Female (ref. Male)*	0.296	<0.001	1.345	1.222–1.479
*Age (ref. ≤34 years old)*				
35–44	0.018	0.773	1.019	0.899–1.154
≥45	0.136	0.132	1.145	0.960–1.366
*Educational level (ref. Associate degree)*				
Bachelor degree	0.031	0.469	1.032	0.948–1.123
Master degree or more	0.041	0.733	1.042	0.822–1.322
*Marital status (ref. Married)*				
Single	−0.027	0.635	0.974	0.873–1.086
Divorced or widowed	0.359	0.003	1.432	1.128–1.817
*Technical post title (ref. Junior title)*				
Intermediate grade	0.060	0.203	1.062	0.968–1.166
Senior title	−0.015	0.857	0.985	0.838–1.159
*Profession (ref. Doctor)*				
Nurse	0.223	<0.001	1.250	1.126–1.388
Medical technicians	0.101	0.053	1.107	0.999–1.226
*Income (ref. ≤3,000 RMB)*				
3,001–8,000	−0.151	0.005	0.860	0.774–0.956
≥8,001	−0.397	<0.001	0.672	0.549–0.823
*Work seniority (ref. ≤10 years)*				
11–20	0.016	0.803	1.016	0.899–1.148
≥21	−0.202	0.025	0.817	0.685–0.974
*Work hours per day (ref. ≤8 h)*	0.536	<0.001	1.710	1.583–1.846
*Experience of workplace violence (ref. Yes)*	−0.584	<0.001	0.558	0.515–0.604

Table 3 displays the references of the categorical variables and the association between anxiety and other factors. Anxiety among primary healthcare workers was associated with female gender (OR = 1.338, 95%CI = 1.198–1.494), being divorced or widowed (OR = 1.373, 95%CI = 1.056–1.770), being a nurse (OR = 1.139, 95%CI = 1.013–1.282), and working more than 8 h per day (OR = 1.638, 95%CI = 1.497–1.794). More than 21 years of working experience (OR = 0.721, 95%CI = 0.6587–0.884) and without experience of workplace violence (OR = 0.539, 95%CI = 0.495–0.587) were protective factors.

**Table 3:** Multiple logistic regression examining individual characteristics associated with anxiety in primary healthcare workers.

Variables	B	*p*-value	OR	95% CI
*Female (ref. Male)*	0.291	<0.001	1.338	1.198–1.494
*Age (ref. ≤34)*				
35–44	−0.031	0.663	0.970	0.844–1.114
≥45	0.027	0.795	1.027	0.839–1.258
*Educational level (ref. Associate degree)*				
Bachelor degree	0.049	0.316	1.050	0.954–1.156
Master degree or more	−0.002	0.989	0.998	0.766–1.301
*Marital status (ref. Married)*				
Single	0.044	0.475	1.045	0.926–1.180
Divorced or widowed	0.317	0.014	1.373	1.056–1.770
*Technical post title (ref. Junior title)*				
Intermediate grade	0.093	0.086	1.097	0.987–1.220
Senior title	0.085	0.370	1.088	0.905–1.309
*Profession (ref. Doctor)*				
Nurse	0.130	0.030	1.139	1.013–1.282
Medical technicians	0.010	0.865	1.010	0.896–1.139
*Income (ref. ≤3,000 RMB)*				
3,001–8,000	−0.174	0.004	0.840	0.747–0.945
≥8,001	−0.280	0.017	0.756	0.601–0.950
*Work seniority (ref. ≤10 years)*				
11–20	−0.036	0.609	0.965	0.842–1.106
≥21	−0.328	0.002	0.721	0.587–0.884
*Work hours per day (ref. ≤8 h)*	0.494	<0.001	1.638	1.497–1.794
*Experience of workplace violence (ref. Yes)*	−0.618	<0.001	0.539	0.495–0.587

## Discussion

### Prevalence of depression and anxiety, and associated factors among primary healthcare workers

In this study, based on a large-scale, cross-sectional study, we primarily focused on depression and anxiety and their correlates. Our survey findings showed that 50.7% of primary healthcare workers scored above the PHQ-9 clinical cut-off score of 5, and 26.4% scored above the GAD-7 clinical cut-off score of 5. The prevalence of depression and anxiety in our study was higher than that among primary healthcare workers during the COVID-19 pandemic in Japan (depression: 15.0%; anxiety: 31.9%) (36), New York City (depression: 33.8%; anxiety: 48.2%) (22) and Colombia (depression: 26.85%; anxiety: 43.19%) (37). In the post-epidemic era, depression is more severe than anxiety among primary healthcare workers. A potential explanation is the selective dynamic temporal interplay between the COVID-19 pandemic and negative emotions. At the onset of the COVID-19 pandemic outbreak, the outbreak acted as a major stressor and significantly increased anxiety among primary healthcare workers, while the impact on depression among primary healthcare workers may take more time (9, 25).

More than two-thirds (72.2%) of primary healthcare workers with depression were female, and 73.3% of primary healthcare workers with anxiety were female. We found that the prevalence of depression and anxiety was both relatively high among some subgroups, such as female nurses (depression: 54.6%; anxiety: 29.2%), females who were divorced or widowed (depression: 60.3%; anxiety: 33.2%), and those with experience of workplace violence (depression: 64.0%; anxiety: 38.0%). Using a regression analysis, we also identified a few important work-related factors that were significantly associated with depression and anxiety in the whole sample. Among the risk factors that may increase the likelihood of developing depressive and anxiety symptoms among primary healthcare workers during the post COVID-19 pandemic, associated factors at the individual level have been highlighted such as female gender, being divorced or widowed, being a nurse, daily work hours, monthly income, working experience and experience of workplace violence.

### Relationship between gender, profession, and depressive and anxiety symptoms

In correspondence, these study results reflect similarities with the already established gender and profession gap findings, suggesting that female healthcare workers were more vulnerable to mental and physical health problems during the pandemic than male healthcare workers (38–40). Our findings suggest that one of the main factors predisposing to depressive and anxiety symptoms could be female sex. This could be justified by the fact that female gender was easy to recognize depression and anxiety, as well as by demographic factors, social, and biological factors, as supported by previous studies (15, 41–43). Serious threats to the mental health of female healthcare workers had equally serious implications for the patients who relied on female healthcare workers in their personal roles as caregivers (38).

In addition, we performed multiple logistic regression analyses to examine the associations between depression/anxiety and other factors (Tables 2, 3), and our results showed that working as a nurse is one of the risk factors for an increase in the levels of depression and anxiety. Moreover, the unpredictable pressure exerted by the COVID-19 pandemic on healthcare systems brings big challenges to nurses, which may affect their mental health well-being (32, 44). Furthermore, several other studies conducted during the initial and ongoing phase of the COVID-19 pandemic also reported similar findings (23, 45). Nurses, as an eminently female profession, were also a higher risk group, and the results highlight the importance of addressing female nurses’ mental health (28, 43).

### Relationship between monthly income, marital status, and depressive and anxiety symptoms

All of these depressive and anxiety symptoms were further exacerbated by variables other than the high workload generated by the COVID-19 pandemic, such as monthly income and marital status. Monthly income was also a documented factor in mental health (46). Primary healthcare workers with a monthly income above RMB 8000/− showed better mental health outcomes as they experienced less depression (297, 4.6%) and anxiety (163, 4.8%). A higher monthly income could bring a sense of security in life and work. This suggests an important role of income as a protective mechanism for mental health in primary healthcare workers, possibly reflecting the fact that individuals with lower income may experience higher financial stress as a consequence of the pandemic (47). Another study conducted in Pakistan by Ullah I et al. indicated that Pakistani healthcare workers having a monthly income of above PKR 100000/− showed worse mental health outcomes in all dimensions of interest, as they experienced more anxiety and stress during the COVID-19 pandemic (15). We hypothesized that higher incomes may be associated with a greater workload and other economic reasons, which may lead to negative emotions (48).

Indeed, a stable social support network had proven crucial for healthcare workers in coping under these strenuous circumstances (49), and a lower level of social support was associated with a greater risk of adverse psychological outcomes (28, 50). Our study suggested that being divorced or widowed by primary healthcare workers was correlated with a higher level of depression and anxiety. Compared with primary healthcare workers who were single and being married, being divorced or widowed by primary healthcare workers who could not get enough family support and share their mental burdens through communication and emotional support turned out to be a more important factor in differentiating depression and anxiety (36). Previous studies have indicated that family and social support, including support from friends and colleagues, may help alleviated feeling of insecurity and loneliness and reduce their depression and anxiety during the post-pandemic period (44, 51). Additionally, married individuals who have strong social support experience a buffering effect on the exacerbation of depression caused by disrupted biological rhythms. This effect is observed across different age groups (52). On the other hand, divorced and widowed individuals may struggle to effectively regulate negative emotions during the COVID-19 pandemic due to the emotional trauma they have experienced in their past marriages (50).

### Relationship between working years, work hours, and depressive and anxiety symptoms

Previous studies have also shown a relationship between work experience and negative emotions, while the factor of working years has shown discrepancies among the different studies (15, 53). The findings suggest that fewer working years were significantly associated with the mental health status of healthcare workers, which was consistent with the findings of the study in Pakistan (15). That is, less work experience was found to be a risk factor for depressive and anxiety symptoms. According to Ullah I et al. (15), younger healthcare workers who had less working experience and were working as frontline forces had worse mental health outcomes. The participants aged between 20 and 24 years old experienced more depression and anxiety in contrast to healthcare workers aged >30 years old. Furthermore, several other studies conducted during the COVID-19 pandemic also represent similar findings that younger healthcare workers working as frontline force having less work experience had experienced more psychological distress (28, 53).

Another variable that generated a higher occurrence of work-related depressive and anxious symptoms in primary healthcare workers was daily work hours. The context of the pandemic had led to an excessive workload, resulting in an increase in depression and anxiety symptoms among healthcare workers (15, 43, 54). The data for this study was collected between 2023/03/01 and 2023/05/31, a timeframe during which our country lifted stringent measures and societal lockdown. As a result of the lifting of the societal lockdown, the number of patients infected with COVID-19 had increased dramatically, and the primary healthcare workers who are at the frontline of this crisis have been facing extreme psychological distress (55). Our findings showed that daily work hours >8 were positively correlated with depressive and anxiety symptoms and were significant predictors of such symptoms. These findings were similar to previous studies, which found that long working hours seemed to be more correlated with physical and psychological problems (45, 56, 57). The irregular and unpredictable nature of working time may contribute to increased levels of psychological distress and challenges in maintaining work-life boundaries (58).

### Relationship between workplace violence and depressive and anxiety symptoms

Furthermore, the findings in this study suggested that higher levels of depression and anxiety among healthcare workers during the post-COVID-19 pandemic era were associated with workplace violence. It was apparent that workplace violence in primary healthcare workers posed a significant risk to their mental health and an occupational health issue of growing concern (59–61). The implications of workplace violence harmed healthcare workers’ psychological and physical well-being (62–64). Victims of workplace violence were more likely to experience depressive symptoms, fear of future workplace violence, poor sleep quality, as well as signs of post-traumatic stress symptoms like direct and vicarious trauma (65, 66). Furthermore, the negative effects of workplace violence on healthcare workers had a significant impact on the quality of care delivered and the turnover intention of healthcare workers (67). The findings of this study could inform the development of support systems to enhance the resilience of healthcare workers experiencing workplace violence by alerting governing institutions. This also leads us to think that the mental health problem of adolescents has become a prominent issue in China at present. School violence, similar to workplace violence, is considered one of the main causes of mental health problems faced by adolescents. Timely intervention on school violence may help solve the anxiety and depression problems of adolescents.

There were a few strengths about this study. First, it was among the first batch to investigate the long-term impact of COVID-19 on psychological symptoms among primary healthcare workers in China during the post-pandemic era. This study included >12,000 healthcare workers working in a primary hospital and having diverse specialties and job descriptions (including the three most important professionals: doctors, nurses, and medical technicians). Second, we used an internationally tested instrument (GAD-7 and PHQ-9) to collect data. This survey was conducted online anonymously, and to encourage honest reporting. Finally, thanks to the support and involvement of primary hospital administrators in Anhui China, the valid response rate was 94.3%, higher than most similar surveys in healthcare workers (23, 36, 68). There were also some limitations, which could be directions for future research. First, the findings of this study are based on self-reports of depressive and anxiety symptoms as opposed to diagnostic criteria or clinical measures. Second, as a cross-sectional survey, it prevents us from making causal claims between depression/anxiety and other factors. Future research using an instrumental variable approach or longitudinal data could better understand the dynamic role of associated factors in shaping depression and anxiety. Third, a limited number of questions included in the questionnaire prevents us from extracting other possible factors that affect the mental health of primary healthcare workers, such as exercise and other health-promoting behaviors. Fourth, due to selection bias caused by our samples, the conclusion could not be generalized to all healthcare workers, particularly those working in tertiary hospitals.

In addition to causing physical diseases, the COVID-19 pandemic has also placed a burden on the mental health of primary healthcare workers that may persist after the pandemic. Health systems should prioritize enhancing the resilience of healthcare workers during the COVID-19 pandemic and recognize the importance of their mental well-being as a global public health priority. These approaches are crucial for effectively addressing the mental health challenges faced by healthcare workers (69, 70).

## Conclusion

Depressive symptoms among primary healthcare workers were highly prevalent during the post-pandemic period. Female gender, being divorced or widowed, being a nurse, work experience, daily work hours, monthly income, and experience of workplace violence were identified as associated factors. Public health prevention programs are needed to prevent and reduce long-term adverse health outcomes and morbidity associated with depressive symptoms.

## Data availability statement

The raw data supporting the conclusions of this article will be made available by the authors, without undue reservation.

## Ethics statement

The studies involving humans were approved by the Ethics Committee of Chaohu Hospital of Anhui Medical University (Approval number #202002-kyxm-02). The studies were conducted in accordance with the local legislation and institutional requirements. The participants provided their written informed consent to participate in this study.

## Author contributions

DL: Methodology, Writing – review & editing. YZ: Data curation, Writing – original draft. XT: Data curation, Investigation, Writing – original draft. YC: Investigation, Writing – original draft. RT: Writing – review & editing.
